# Role of Bubble Evolution in the Bubble-Propelled Janus Micromotors

**DOI:** 10.3390/mi14071456

**Published:** 2023-07-20

**Authors:** Gang Chen, Xuekui Wang, Bingyang Zhang, Fangfang Zhang, Zhibin Wang, Baiqiang Zhang, Guopei Li

**Affiliations:** 1 School of Energy and Power Engineering, Zhengzhou University of Light Industry, Zhengzhou 450002, China; 2 School of Material and Energy, Guangdong University of Technology, Guangzhou 510006, China

**Keywords:** Janus micromotor, bubble growth, collapse, displacement

## Abstract

Bubble-propelled Janus micromotors have attracted extensive attention in recent years and have been regarded as powerful tools in the environmental and medical fields due to their excellent movement ability. The movement ability can mainly be attributed to the periodic growth, detachment, and/or collapse of the bubble. However, subjected to the experimental conditions, the mechanism of bubble evolution on the motion of the micromotor could not be elucidated clearly. In this work, a finite element method was employed for exploring the role of bubble evolution in bubble-propelled Janus micromotors, which emphasized the growth and collapse of bubbles. After the proposed model was verified by the scallop theorem, the influence of the growth and rapid collapse of bubbles on micromotors was investigated. Results show that the growth and collapse of a bubble can drive the micromotor to produce a displacement, but the displacement caused by a bubble collapse is significantly greater than that caused by bubble growth. The reasons for this phenomenon are analyzed and explained. In addition to the influence of bubble size, the collapse time of the bubble is also investigated.

## 1. Introduction

Self-propelled micromotors [1,2], known for moving autonomously in fluids by harvesting energy from the surrounding environment, have been regarded as prospective tools to implement assigned operations in various potential scenarios, ranging from the biomedical to the water purification field [3,4,5,6,7,8]. Research enthusiasm for micromotors has continued for several years, and hence, tremendous advances in understanding the motion principles and carrying out applied research have been achieved. In terms of the movement of the micromotors, the driving force can be derived from the chemical catalytic reaction [9,10] or external stimuli such as thermal [11], ultrasound [12], electric [13], and light [14,15]. Among them, micromotors driven by gas bubbles are certified to reach satisfactory movement speed [16], which has attracted highly popular concern.

For the bubble-propelled microreactors, tubular and spherical microreactors are the two main categories according to the structural differences [17]. Tubular micromotors are generally fabricated by strain-induced self-rolling nanomembranes, template-based electrodeposition, or two-photon laser lithography [18,19,20]. The inner and outside surfaces of the tube usually deposit a catalytic layer (e.g., Pt, Ag, Fe, enzyme) and a functional layer (e.g., Au, Fe_3_O_4_, SiO_2_), respectively, for bubble formation and microreactor guidance, for in situ reactions or for multi-functional purposes [20,21,22,23,24]. The most common method for generating bubbles is the decomposition of H_2_O_2_ into O_2_ under the action of the Pt catalytic layer, the O_2_ bubble growth and ejection from one side of tubular micromotors enables propulsion. By contrast, spherical micromotors with the catalytic layer and functional layer distributed on half the surface of the sphere, decompose the H_2_O_2_ into O_2_ on the hemispherical surface covered with the catalytic layer [25]. Previous studies [26] proved that O_2_ bubbles are generated only when the diameter of spherical micromotors is larger than 10 μm, and the periodic generation of the bubbles and the interaction between the bubble and micromotors induce fast motion.

As we know, sufficiently fast speed and long operational lifetime are essential for micromotors in order to perform specific tasks more efficiently, while this is closely related to bubble behavior [17,27]. Therefore, a good understanding of bubble dynamics is essential and should be given extra attention. The motion mechanism of bubble-propelled micromotors has been explored extensively in recent years [28,29,30,31]. A relatively consistent understanding of the two kinds of micromotors is that the driving force of the bubble acting on the micromotors mainly comes from the direct momentum exchange during bubble growth and recoiling stages. The driving force depends on the diameter and generation/release frequency of the bubbles, which are also associated with the concentration and surface tension of the fuel solution [30,32].

One thing worth noting is that bubble collapse could happen in liquid under some circumstances [33]. At the scale of centimeters and millimeters, the published literature [34,35] has demonstrated that the collapse of the bubble near a rigid wall could cause a micro-jetting flow. For a bubble near a suspended particle with the same scale, the collapse of the bubble was considered to pull the suspended particle toward the center of the bubble. At the micrometer scale, Manjare et al. [29] observed the motional behavior of a bubble-propelled micromotor and considered that the bubble growth process resulted in a growth force moving the micromotor away from the bubble, while the collapse process induced an instantaneous local pressure depression pulling the micromotor toward the bubble. Unfortunately, they did not observe any obvious micro-jetting flow in the experiments. On the contrary, Zheng et al. [36] developed a hollow spherical Pt-SiO_2_ micromotor and found that motion direction and speed were diverse with the dynamic change of the bubble. Compared to the speed during bubble growth and recoiling, a speed of more than three orders of magnitude was observed during the bubble collapse stage. Moreover, after a transient pull back to the bubble’s center, the micromotor rapidly moved away from the bubble. They concluded that the collapse of the bubble induced an impulsive jetting flow that instantaneously pushed the micromotor forward.

It can be seen that the underlying mechanisms responsible for micromotor propulsion are not very explicit and are worthwhile to investigate in detail. Although the experimental studies could capture the bubble’s behavior and even prove the formation of micro-jetting, the interaction between the bubble and micromotors could not adequately explain by the visualization technique. Zheng et al. [37] simulated the flow field and the concentration field near the surface of the micromotor but ignored the influence of the dynamic behavior of bubbles. Zhang et al. [38] found that bubble collapse could cause micro-jetting and promote the movement of micromotors through numerical simulation based on the VOF method. However, the research was limited to the bubble collapse stage, and the influence of periodic bubble evolution on micromotors is still lacking.

In this work, the effects of a complete bubble cycle, which includes bubble growth and bubble collapse, on the motion of a micromotor were studied by a numerical simulation. Firstly, the growth and collapse of the bubble were regarded as reversible reciprocating motions to coincide with the scallop theorem. Then, the influence of growth and rapid collapse of the bubble on the motion of the micromotor was highlighted. The reasons for the distinct displacement of a micromotor caused by two bubble behaviors were analyzed and explained. Finally, the influence of bubble size and collapse time of the bubble was also studied.

## 2. Model and Method

### Physical and Numerical Model

**Figure 1:**
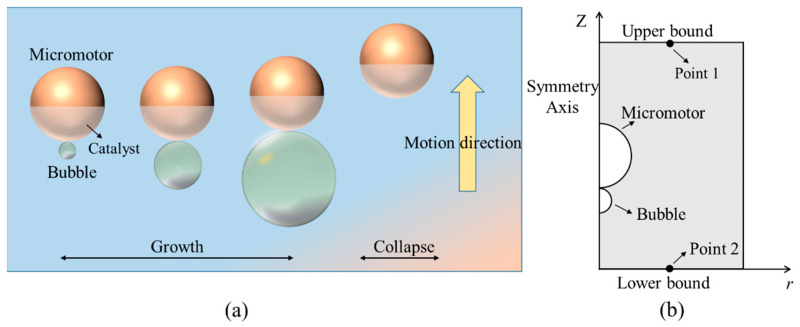
Physical model (**a**) and numerical model (**b**) of bubble-propelled micromotor.

The schematic diagram of a typical bubble-propelled Janus micromotor is shown in Figure 1a. By decomposing the chemical fuels in a solution on the catalyst side, the gas molecules generate and when the dissolved gases reach saturation, a bubble will form on the surface of the micromotor. As time goes on, the bubble continues to grow and expands to its maximum size. During this period, the micromotor moves away from the bubble and the driving force for the micromotor mainly comes from bubble growth. In addition, if the bubble with the maximum size collapses in certain conditions, an obvious displacement away from the bubble will be observed. Actually, for this case, a complete bubble cycle could be divided into three stages, namely bubble growth stage, bubble collapse stage, and bubble post-collapse stage. The influences of bubbles appear in the first two stages, while no visible bubbles appear in the last stage. Researches show that the speed of micromotor caused by bubble collapse is three orders of magnitude more than by bubble growth. Therefore, the growth and collapse of the bubble need to be focused on and explored.

To achieve this goal, a finite element method based on COMSOL Multiphysics was used to simulate the processes of the bubble-propelled micromotor. Because of the low flow velocity, the laminar flow module was used to calculate the flow field in the simulation process. The governing equations are the continuity equation and the N-S equation of incompressible fluid. A two-dimensional axisymmetric model was adopted, as shown in Figure 1b. It should be noted that the bubble has an initial size. The processes of bubble growth and collapse were substituted for a spherical wall with a periodic variable dimension. Based on this, the functions of radius and velocity of the bubble in the growth stage, collapse stage, and post-collapse stage are given, as shown in Equations (1)–(5). *R_bg_*, *R_bc,_* and *R_bp-c_* represent the size of bubble growth as well as the collapse and post-collapse stages, respectively. Accordingly, *V_bg_ and V_bc_* represent the velocity of bubble growth and collapse stage. A fixed distance between the bubble and the micromotor was set in order to ensure the convergence of the calculation. In addition, the simulation of the movement of the Janus micromotor in the fluid needs to set a moving grid on the solid boundary, which was very complicated. Therefore, a relative coordinate system was employed in this paper, in which the Janus micromotor was fixed and the relative motion of the fluid was used to reflect the motion of the Janus micromotor.

(1) $$R_{bg}(t)=-A\cos\left(\frac{2\pi }{T_{g}}t\right)+A+R_{b\min}$$

(2) $$V_{bg}\left(t\right)=A\frac{2\pi }{T_{g}}\sin\left(\frac{2\pi }{T_{g}}t\right)$$

(3) $$R_{bc}(t)=A\cos\left[\frac{2\pi }{T_{c}}\left(t-\frac{T_{g}}{2}\right)\right]+A+R_{b\min}$$

(4) $$V_{bc}(t)=-A\frac{2\pi }{T_{c}}\sin\left[\frac{2\pi }{T_{c}}\left(t-\frac{T_{g}}{2}\right)\right]$$

(5) $$R_{bp-c}(t)=R_{b\min}$$

## 3. Results and Discussion

### 3.1. Model Validation by the Scallop Theorem

In this study, bubble growth and collapse were mainly considered. Because of the inherent limitations of the model, an initial bubble size was set and the processes of bubble growth and collapse were substituted for a spherical wall with a periodic variable dimension. Namely, the bubble grows to its maximum size from the initial size during the growth stage and collapses to the initial size from the maximum size during the collapse stage. Therefore, the bubble growth and collapse can be regarded as reciprocating motion, which is an essential element of the scallop theorem. As we know, the scallop theorem states that when an object undergoes reversible reciprocating motion in an incompressible Newtonian fluid at a low Reynolds number (Re number), its net displacement must be zero. There are three key points in the scallop theorem: a low Re number, an incompressible Newtonian fluid, and reversible reciprocating motion. These conditions are easy to carry out in the proposed model, therefore, if the scallop theorem can be validated, it shows that the proposed model is reasonable.

However, the actual situation is that the bubble grows slowly and collapses rapidly. The rapid collapse of the bubble might cause micro-jetting and push the Re number high. As a consequence, inertial forces can be introduced and thus the related flow could not be regarded as low-Reynolds number Stokes flow. Therefore, to meet the condition of a low Re number in this section, the cycle of growth and collapse remains the same. The detailed parameters are shown in Table 1. It is worth noting that bubble generation depends closely on the geometry of spherical Janus micromotors. Janus micromotors with a large enough diameter can be propelled by bubbles, but bubble propulsion for Janus micromotors less than 10 μm does not occur readily. Bubble nucleation on spherical Janus nanomotors can reliably be observed only if their size is above a certain critical value [39]. In addition, Zheng et al. [26] observed the bubble behavior generated on the micromotor with a diameter of 33.2 μm and demonstrated the micromotor’s motion characteristics. For convenience, the radius of the micromotor was defined as 33 μm. The purpose of this paper is mainly focused on the influence of growth and rapid collapse of bubbles on micromotors; thus, the simulated solution is water. In addition, a relative coordinate system was employed in the model, in which the Janus micromotor was fixed and the relative motion of the fluid was used to reflect the micromotor’s motion, thus the defined materials of the Janus micromotor were set to be a solid wall.

**Figure 2:**
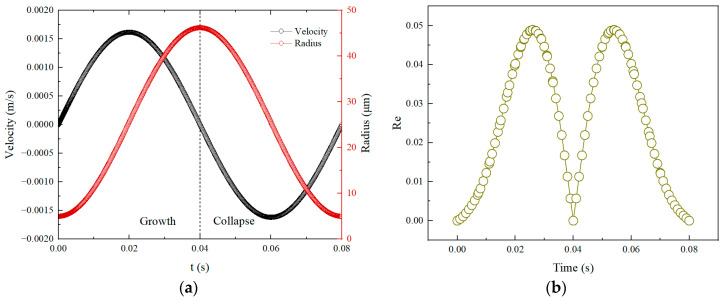
(**a**) Variation of velocity and radius of bubble versus time. (**b**) Variation of Re number versus time.

Variations of velocity and radius of the bubble with time are shown in Figure 2a; the radius curve is axisymmetric with the time of 0.04 s as the axis of symmetry. The bubble reaches its maximum size after 0.04 s of growth from the minimum size of 5 μm. Then, it enters the collapse stage and decreases to the minimum size. The velocity curve of the bubble demonstrates that the velocity of bubble growth is positive when *t* < 0.04 s, and the collapse velocity is negative when *t* > 0.04 s. Therefore, the bubble growth and collapse are symmetrical and the shape is reversible. Figure 2b shows the variation of the Re number in a complete bubble cycle versus time. It can be seen that the Re numbers are always small and thus the related flow belongs to low Re-numbers Stokes flow.

**Figure 3:**
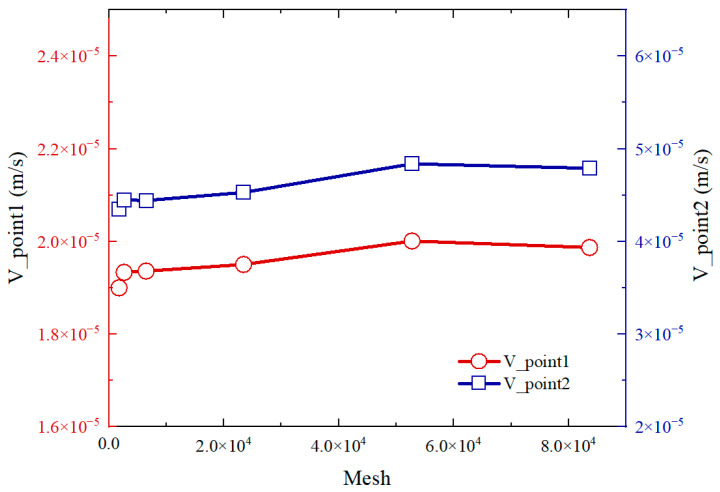
Grid independence testing by the velocities of two different observation points.

**Figure 4:**
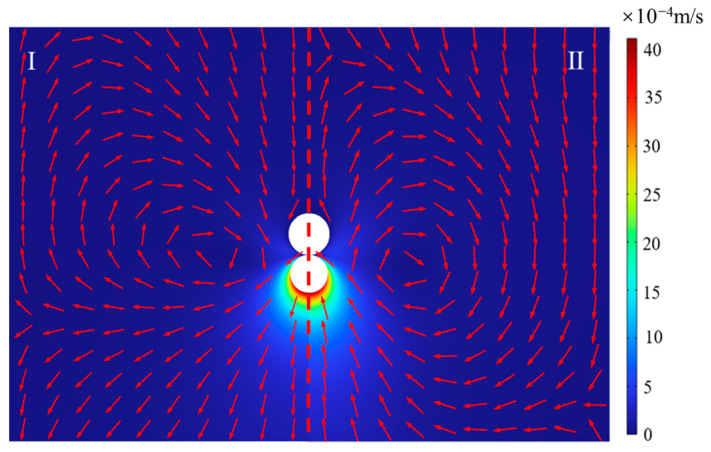
The flow field caused by bubble growth and collapse with the same bubble size. Left I: bubble growth stage, *t* = 0.028 s. Right II: bubble collapse stage, *t* = 0.052 s.

Figure 3 shows the variation of the average velocity of the midpoint of the upper boundary (point 1) and lower boundary (point 2) with different grid quantities. As can be seen, the curves are gradually steady and the error gradually decreased with the increase of the number of grids. More grids will cause a longer calculation time. Therefore, the grid of 52,760 is chosen in this paper. Figure 4 shows the flow field caused by bubble evolution, which is merged into two regions. Region I represents the flow field caused by bubble growth when *t* = 0.028 s, while region II represents the flow field caused by bubble collapse when *t* = 0.052 s. Both of them have the same bubble size of 60 μm. The color and the directions of the red arrows in the picture mean the magnitude and directions of the velocity, respectively. As can be seen, the velocity at the bottom of the bubble is equal in magnitude and opposite in direction, this is because the cycle of bubble growth and collapse is the same. Meanwhile, the bubble pushes the fluid outward during its growth stage while the fluid flows to the bubble during the bubble collapse stage. Moreover, the maximal velocities of the flow field in the magnitude of 10^−3^ are observed, which can also demonstrate low Reynolds number flow.

The motion of a micromotor will cause a change in boundary flow, and reversely, the boundary flow could be used to reflect the motion of the micromotor in relative coordinates. For the sake of simplification, the flow variations are discussed and the obtained rules are qualitatively consistent with the motion of the micromotor. In the model, *q*1 and *Q*1 are defined as the instantaneous flow and total flow through the upper boundary, respectively. While *q*2 and *Q*2 are defined as the instantaneous flow and total flow through the lower boundary, respectively. Meanwhile, the fluid flows in and out from the upper boundary are defined as negative and positive, respectively. The lower boundary is defined the same way but the values are reversed.

**Figure 5:**
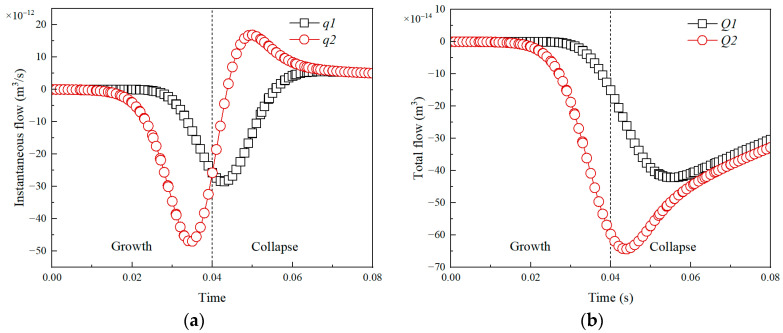
Instantaneous flow (**a**,**b**) total flow of the boundary caused by the bubble growth and collapse. *q*1 and *Q*1: Upper bound, *q*2 and *Q*2: Lower bound.

**Figure 6:**
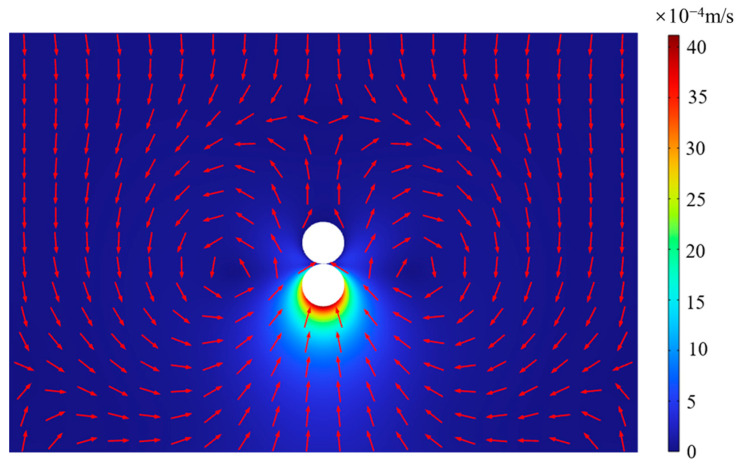
The flow field caused by bubble collapse when *t* = 0.05 s.

Figure 5 demonstrates the variation of the instantaneous and total flow of boundary versus time in a complete bubble cycle. It can be seen that from Figure 5a, the instantaneous flow of both the upper and lower boundaries is negative during the bubble growth phase, meaning that fluid flows in from the upper boundary and out from the lower boundary. The instantaneous flow of upper and lower boundaries becomes positive during the bubble collapse phase, meaning that fluid flows in from the lower boundary and out from the upper boundary. Yet, the instantaneous flow of the lower boundary changes to a positive value faster than that of the upper boundary. It can be explained by Figure 6. As seen, the flow directions of the upper boundary and lower boundary are downward and upward, respectively, which means the instantaneous flow of the upper and lower boundary is negative and positive. In addition, the instantaneous flow of the lower boundary is always larger than that of the upper boundary. It may be the bubble is closer to the lower boundary and meanwhile the Janus micromotor has a certain blocking effect on fluid flow.

Figure 5b shows the variation of the total flow of the boundary versus time in a complete bubble cycle. As can be seen, the total flow of the upper and lower boundary is always negative, which means bubble evolution causes the fluid to flow in from the upper boundary and out from the lower boundary. At the end of a bubble growth period, the total flow (termed as *Q_tg_*) is −4.5 × 10^−13^ m^3^. At the end of a complete bubble period, the total flow (termed as *Q_t_*) is almost −3.3 × 10^−13^ m^3^. Here, a relative flow is defined as η = abs (*Q*/*V_b_*_max_), where *V_b_*_max_ (4.1 × 10^−13^ m^3^) is the maximum bubble volume. Thus, the value of η is 1.1 and 0.8, respectively, for the bubble growth and the complete bubble period (growth and collapse). That is, the volume flow caused by bubble growth can drive a volume flow of 1.1 times the bubble volume, while the volume flow caused by the complete bubble period only can drive a volume flow of 0.8 times the bubble volume. Namely, a single bubble growth can drive the micromotor to produce a net displacement, while a completely reversible reciprocating bubble cycle could not cause the micromotor to produce a displacement. The obtained results are consistent with the scallop theorem and demonstrate the correctness of the numerical calculation model.

### 3.2. The Growth and Rapidly Collapse of Bubble

In fact, the bubble grows slowly and takes tens of milliseconds to reach the maximum diameter, while the bubble collapses rapidly and only takes ten microseconds. In the post-bubble collapse stage, the fluid still has velocity under the action of bubble deformation in the first two stages, and the velocity gradually decreases, and returns to the state before the growth of the bubble. To simulate the growth and rapid collapse of the bubble, the bubble collapse cycle is set to 0.2 ms, and the other settings are the same as those in Table 1.

**Figure 7:**
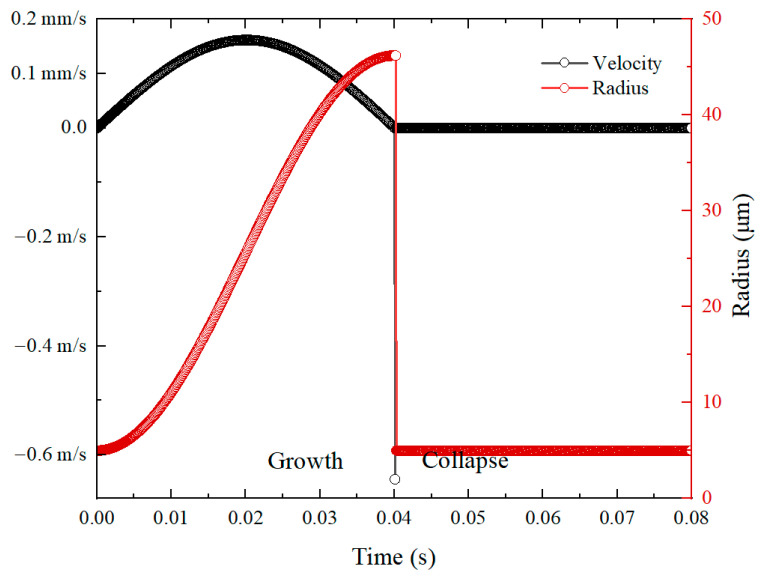
The variation of velocity and radius versus time.

Figure 7 shows the variation in velocity and radius of the bubble versus time. It can be seen that when the bubble goes into the collapse stage, the radius rapidly decreases to the minimum value. Meanwhile, during the bubble growth stage, the velocity is at the magnitude of ~mm/s, while during the collapse stage, the velocity is at the magnitude of ~m/s. The velocity in the bubble collapse stage is three orders of magnitude larger than that in the growth stage. Therefore, the Re number in the bubble collapse stage is three orders of magnitude higher than that in the growth stage. In this situation, the inertia force cannot be ignored.

**Figure 8:**
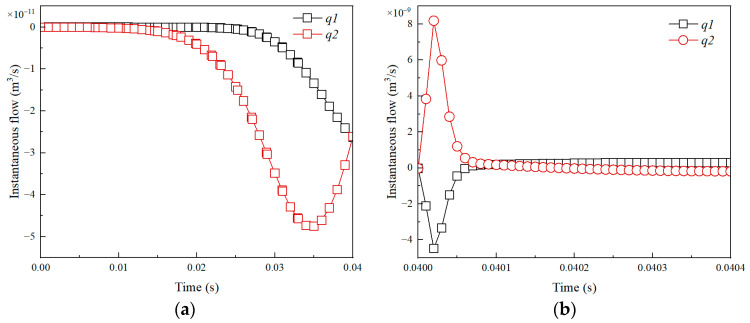
Instantaneous flow of boundary caused by bubble growth (**a**,**b**) collapse. *q*1: Upper bound; *q*2: Lower bound.

**Figure 9:**
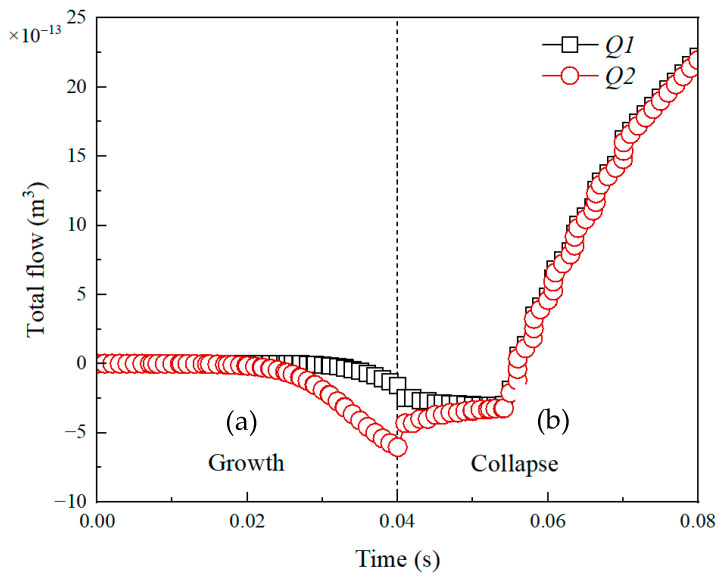
The total flow of the boundary caused by the bubble growth (**a**,**b**) collapse. *Q*1: Upper bound; *Q*2: Lower bound.

The instantaneous flow of upper and lower boundaries is shown in Figure 8a. It can be seen that in the bubble growth stage, the boundary flow rate is about 10^−11^ m^3^/s. In the bubble collapse stage, the boundary flow rate is about 10^−9^ m^3^/s. In the stage of bubble post-collapse, the volume flow rate gradually tends to 0. The results in Figure 9b shows that at the end of a bubble period, the total flow is 2.3 × 10^−12^ m^3^. Thus, the relative flow η is 5.6, which indicates the Janus micromotor will have a significant net displacement.

### 3.3. The Factors Affecting the Motion of Janus Micromotor

**Figure 10:**
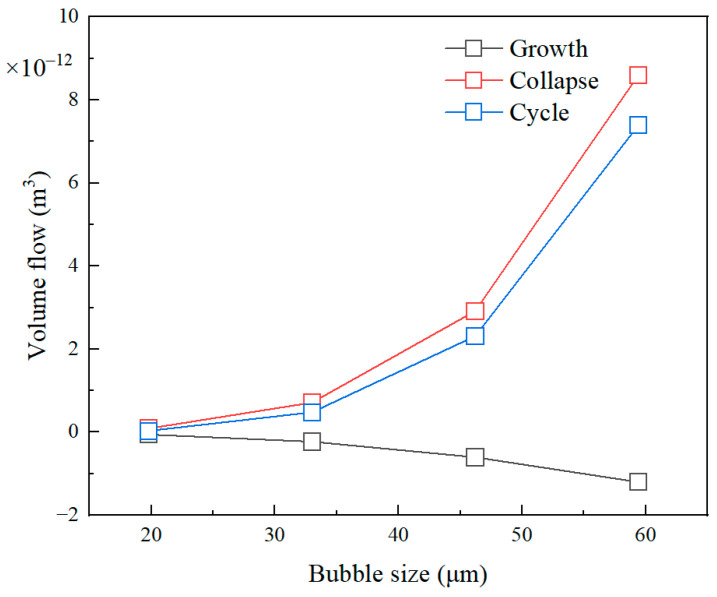
The relationship of volume flow and bubble size during different bubble stages.

Because the motion of the micromotor mainly depends on the bubble behavior and the surrounding environment, the influence of bubble size and collapse time of the bubble on the micromotor is investigated in this section. The variations of volume flow with the bubble size during different stages are shown in Figure 10. Here, the maximum bubble size is set to be 0.6, 1, 1.4, and 1.8 times the size of the micromotor. It can be seen that with the increase of bubble size, the volume flow (in terms of absolute size) during the bubble growth stage, bubble collapse stage, and a bubble growth-collapse cycle are all increased. A larger volume flow means a larger net displacement of a micromotor; thus, the increase in bubble size contributes to the movement of micromotors. It’s worth noting that, the volume flow during the bubble collapse stage obviously exceeds that during the bubble growth stage. Moreover, the increasing amplitude in volume flow is becoming increasingly obvious with the increase in bubble size. The reasons can be ascribed to the fact that the rapid collapse of the bubble increases the local Reynolds number, and the introduced inertial force makes an increase in volume flow. In addition, a larger bubble size means a larger bubble volume and more fluid could be driven as it grows. Meanwhile, when a bubble with a large size collapses, it may produce more energy. Therefore, making the bubble size as large as possible and making the bubble collapse quickly should be an effective way to promote the motion of the micromotor.

**Figure 11:**
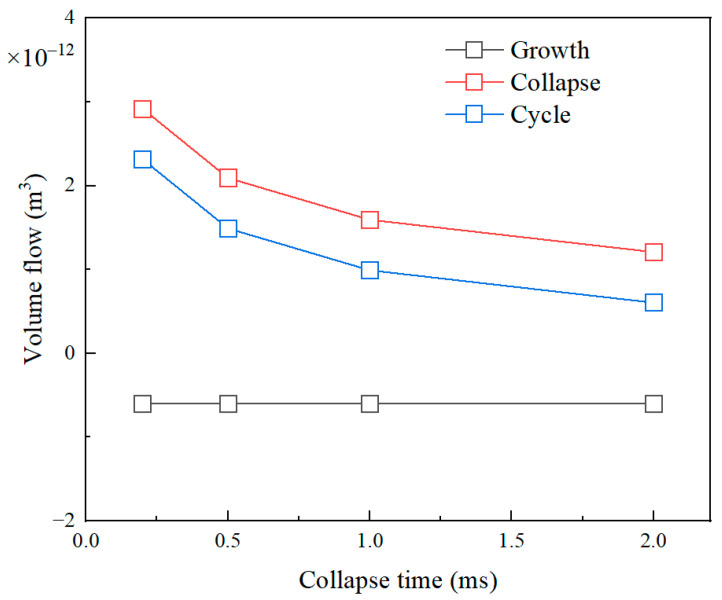
The relationship of volume flow and bubble collapse time during different bubble stages.

The collapse time of the bubble is also an important factor to influence the flow field, furthermore the volume flow. The variations of volume flow with the collapse time of the bubble during different stages are shown in Figure 11. The collapse time of the bubble was set to 0.2 ms, 0.5 ms, 1 ms, and 2 ms, respectively. It can be seen that the volume flows during the bubble stage and a bubble growth-collapse cycle decrease with the increase of collapse time. During the bubble growth stage, the volume flow is constant. This is because the growth time and maximum size of the bubble are fixed, so the collapse time of the bubble does not affect the growth stage. In addition, the increased collapse time of the bubble means a decrease in collapse speed, and the influence of introduced inertia on the flow field is weakened. Therefore, the net displacement of the micromotor will decrease with the increased collapse time of the bubble.

## 4. Conclusions

In this work, a finite element method was employed for exploring the role of bubble evolution in the bubble-propelled Janus micromotor. The effects of bubble growth and collapse on the motion of the micromotor are focused. Results show that when the bubble undergoes a reversible reciprocating deformation in an incompressible Newtonian fluid at a low Reynolds number, the displacement of the micromotor was zero, which is consistent with the scallop theorem. Then, the influence of growth and rapid collapse of the bubble on the micromotor is highlighted. Results show that the growth and collapse of the bubble can drive the micromotor to produce a net displacement, but the displacement produced by the latter is far larger than that by the former. The phenomenon that the micromotor is driven more efficiently by bubble collapse is explained rationally. The rapid collapse of the bubble will cause the Reynolds number to increase significantly, and thus the introduced inertial force will drive the micromotor to produce an obvious displacement. In addition to the influence of maximum bubble size, the collapse time of the bubble was also studied. Results show that a larger bubble size and a shorter collapse time of the bubble contribute to the larger displacement of the micromotor. This paper not only gives a new insight into how to reveal the impelling action of a bubble on a micromotor but also points out the directions to enhance the micromotor’s motion characteristics.

## Figures and Tables

**Table 1:** Relevant parameters of numerical calculations.

Title	Value	Description
*R_j_*	3.3 × 10^−5^ m	Radius of Janus micromotor
*R_b_*_min_	5 × 10^−6^ m	Minimum radius of the bubble
*H*	6.6 × 10^−4^ m	Height of computational region
*W*	4.95 × 10^−4^ m	Width of computational region
*d*	3.3 × 10^−8^ m	Distance of micromotor and bubble
*h_b_*	2.9197 × 10^−4^ m	Height of the center of the bubble
*h_j_*	3.3 × 10^−4^ m	Height of the center of micromotor
*T_g_*	0.08 s	Cycle of the bubble growth
*T_c_*	0.08 s	Cycle of the bubble collapse
*T_p-c_*	0 s	Cycle of the bubble post-collapse
*R_b_*_max_	4.62 × 10^−5^ m	Maximum radius of bubble
*A*	2.06 × 10^−5^ m	Amplitude of a periodic function

## Data Availability

The data that support the findings of this study are available from the corresponding author upon reasonable request.
